# Genetic variability of blainvillea yellow spot virus (*Begomovirus blainvilleae*) reveals recombinant variants and a distinct nonanucleotide motif

**DOI:** 10.1007/s00705-026-06689-z

**Published:** 2026-07-20

**Authors:** Marcelo H. O. Gonçalves, Ayane F. F. Quadros, Mayra M. M. Ferro, Anelise F. Orílio, João Paulo H. Silva, Julia B. Lage, Sarah J. C. Silva, F. Murilo Zerbini

**Affiliations:** 1 Departamento de Fitopatologia/BIOAGRO, Universidade Federal de Viçosa, Viçosa, MG 36570-900 Brazil; 2 Setor de Fitossanidade, Campus de Engenharias e Ciências Agrárias, Universidade Federal de Alagoas, Rio Largo, AL 57100-000 Brazil

## Abstract

Viruses from wild plants may emerge as new economically important agricultural pathogens through spillover events. Blainvillea yellow spot virus (*Begomovirus blainvilleae*, BlYSV) is a bipartite geminivirus which is ubiquitous in the non-cultivated plant *Blainvillea rhomboidea*. To better understand the ecological and evolutionary aspects of this virus, we sampled *B. rhomboidea* in three Brazilian states between 2020 and 2023. Following rolling-circle amplification, cloning and Sanger sequencing, a dataset was assembled with 23 complete DNA-A sequences plus an additional set of 30 BlYSV sequences retrieved from GenBank. Four variants (named A to D) were identified based on sequence comparisons, one of which (variant A) was classified as a different strain. A recombination event in variant B isolates, followed by positive selection in their *AC4* gene, may have increased the fitness of these isolates, which replaced variant C isolates in Viçosa and Coimbra between 2014 and 2022. Four sequences in variant A exhibited a nucleotide substitution (A2667G) within the geminivirus conserved nonanucleotide motif (from TAATATTAC to TAATGTTAC). Our results confirm the high genetic variability of this virus which was hinted at in previous studies. Future studies will be directed at understanding the impact of the observed mutations in viral fitness, and whether this could facilitate spillover of BlYSV to other hosts, including cultivated plants.

**Supplementary Information:**

The online version contains supplementary material available at 10.1007/s00705-026-06689-z.

## Introduction

The ability of viruses to evolve at a faster rate than observed in other organisms allows them to rapidly adapt to environmental changes and exploit new niches [1]. The study of virus populations in non-cultivated hosts is relevant from both basic and applied perspectives: unmanaged ecosystems allow the study of virus evolution in the absence of cultivation-related bottlenecks, and introduced crops may be infected by “indigenous” viruses present in non-cultivated hosts [2].

The family *Geminiviridae* includes plant viruses characterized by twinned quasi-icosahedral particles and a single-stranded, circular DNA genome [3]. These viruses are noted for their high evolutionary rates, similar to those of RNA viruses [4]. Currently, 15 genera and over 500 species are recognized by the International Committee on Taxonomy of Viruses (ICTV), with new species being continuously reported [5]. Most of these species belong to the genus *Begomovirus* and are naturally transmitted by whiteflies of the *Bemisia tabaci* cryptic species complex. The majority of begomoviruses found in the Americas (AM) have two genomic components, named DNA-A and DNA-B, each approximately 2,600 nucleotides (nt) long. In contrast, begomoviruses in Europe, Africa, Asia and Oceania (EAAO) may be mono- or bipartite [6].

Typical AM bipartite begomoviruses have five genes in the DNA-A (*CP*, *Rep*, *REn*, *TrAP* and *AC4*) involved in viral replication, particle formation, and suppression of RNA silencing. The DNA-B, in turn, encodes two proteins (NSP and MP) associated with nuclear transport and systemic movement in the plant. The two segments do not share sequence identity except for an intergenic region, referred to as the common region (CR), which is variable among different species but highly conserved in the DNA-A and DNA-B of the same species [7]. This region includes a hairpin structure with a nonanucleotide motif (5’-TAATATTAC-3’) in the loop region, which is the origin of rolling-circle viral replication [8]. This nonanucleotide is highly conserved across the *Geminiviridae*, except in the genera *Becurtovirus*, *Eragrovirus*, and one member of the genus *Mastrevirus*, which harbor the alternative sequence 5’-TAAGATTCC-3’ [9, 10]. Furthermore, two begomoviruses exhibit a nonanucleotide similar to those found in members of the families *Alphasatellitidae* and *Nanoviridae* (5’-TAGTATTAC-3’), likely acquired through recombination [11, 12].

Brazil is recognized as a center of begomovirus diversity, and the genetic diversity and variability of these viruses have been extensively studied over the years in both cultivated and non-cultivated hosts [5, 13–20]. These studies indicated that non-cultivated plant species of the genera *Sida* and *Macroptilium* act as “mixing vessels”, showing high permissibility to mixed infections with a diverse array of viruses that often recombine, conditions that are thought to favour spillover events to cultivated plants [2]. Other species, such as *Cleome affinis* and *Euphorbia heterophylla*, act as “sealed containers”, harboring almost exclusively a single begomovirus which, in turn, is rarely if ever found in other hosts.

*Blainvillea rhomboidea* exhibits a sealed container relationship with Blainvillea yellow spot virus (*Begomovirus blainvilleae*, BlYSV), currently the only begomovirus known to infect this host [15]. A study that assessed the genetic variability and population structure of tomato-infecting begomoviruses in Brazil also included a small number of BlYSV isolates, and the analyses indicated that this virus has a significantly higher degree of genetic variability compared to the tomato-infecting begomoviruses [13]. Interestingly, a more recent study analyzing the complete genome (DNA-A and DNA-B) of a larger number of isolates from distant geographical regions found that, unlike most begomoviruses analyzed to date and despite their high genetic variability, BlYSV isolates shown no evidence of population subdivision or geographical segregation [15]. A more detailed study of BlYSV may provide useful insights related to the evolution of viral populations in sealed container hosts.

The objective of this study was to characterize the genetic variability and evolutionary mechanisms acting upon BlYSV populations.

## Methods

### Sample collection

 Samples of *Blainvillea rhomboidea* and *Phyllanthus niruri* plants exhibiting symptoms of viral infection were collected from the municipalities of Viçosa, Coimbra, Muriaé (state of Minas Gerais, MG), Touros (state of Rio Grande do Norte, RN), Rio Largo and Maceió (state of Alagoas, AL) between 2020 and 2023 (Suppl. Table S1).

### Cloning and sequencing of begomovirus genomes

 Total DNA was extracted from leaf samples as described [21]. The viral genome was amplified by rolling circle amplification (RCA) [22] and was digested with *ApaI*, *HindIII*, *PstI* and *XbaI*, chosen with the assistance of A Plasmid Editor (ApE) software (https://jorgensen.biology.utah.edu/wayned/ape/) to selectively cleave the DNA-A component of BlYSV at a single site. Fragments of approximately 2,600 nt were cloned into the pBluescript KSII(+) vector, which had been previously digested with the corresponding enzyme and dephosphorylated. Recombinant plasmids were transformed into *Escherichia coli* DH5α by electroporation, and completely Sanger-sequenced at Macrogen (Seoul, Republic of Korea). Genome assembly was performed using SeqAssem (https://science.do-mix.de/software_seqassem.php), and annotation was conducted using ORFfinder (https://www.ncbi.nlm.nih.gov/orffinder/). Species identification was confirmed by pairwise comparisons using SDT v. 1.3 [23] against DNA-A sequences of BlYSV retrieved from GenBank (Suppl. Table S2). A threshold of 91% nucleotide (nt) sequence identity was used to delineate individuals of the same species, and 94% nt identity was used for the same strain, as per the guidelines of the *Geminiviridae* and *Tolecusatellitidae* Study Group of the ICTV [6]. An identity threshold of 97% was arbitrarily applied for the demarcation of BlYSV variants. The Mfold web server [24] was used to computationally predict the secondary structure in the nonanucleotide motif with lowest ΔG value.

### Intraspecific recombination and phylogenetic analyses

 A multiple sequence alignment was obtained with the MAFFT algorithm [25] on an online server, and recombinant events were examined using RDP5 [26]. Each method implemented in RDP5 was configured with default parameters, and significance was determined with a Bonferroni-corrected *p*-value of 0.05. Only recombination events identified by a minimum of four methods were considered reliable. The General Time Reversible nucleotide substitution model with gamma distribution and invariant sites (GTR + G+I) was selected using MrModelTest [27]. This model was then used to construct a Bayesian phylogenetic tree using the MrBayes program [28] available on the CIPRES server [29]. The tree was generated through two independent runs of 10,000,000 generations sampled at every 1,000 generations, and was visualized and edited using Figtree (https://tree.bio.ed.ac.uk/software/figtree/). Bean golden mosaic virus (*Begomovirus costai*, BGMV) served as an outgroup in a neighbor-joining analysis with 1,000 bootstrap replications using MEGAX [30] to determine the root of the Bayesian phylogenetic tree. In addition to the phylogenetic analysis of the complete DNA-A segment, independent Bayesian phylogenetic analyses were performed for the *Rep* and *CP* genes to assess the congruence between the two ORFs. The presence of a temporal signal within the population was evaluated with the TempEst program [31], by correlating the root-to-tip genetic distance with the year of collection of the isolates.

### Population structure and genetic diversity

 Multivariate statistical analysis was conducted using Discriminant Analysis of Principal Components (DAPC) with the adegenet package [32] implemented in R software. Subpopulations were defined using the *k*-means clustering approach, with the number of clusters (*k*) ranging from 1 to 10 to maximize the variation between groups. The optimal number of subpopulations was determined using the Bayesian Information Criterion (BIC). The optimal principal component values were investigated to assess the proportion of successful reassignment, a-score and cross-validation. To support the results, the Nst statistic, analogous to Wright’s Fixation index at the nt sequence level, was estimated between subpopulations and within each one using DnaSP v. 6 [33]. The main descriptors of genetic variability (haplotype number, haplotype diversity, nucleotide diversity, mutation rate, total number of polymorphic sites, total number of mutations, and average number of nucleotide differences) were evaluated using DnaSP. The statistical significance of the differences in π values between subpopulations was assessed as described [34].

### Selection pressures

 Evolutionary mechanisms in individual ORFs at the population and subpopulation levels were investigated using Tajima’s D [35], Fu and Li’s D* and F* tests [36], conducted in DnaSP. These tests were performed under the assumption of a null hypothesis of neutrality. Only *p*-values > 0.05 were considered sufficient to accept the null hypothesis. Selection pressures were assessed based on the dN/dS ratio. Sites subject to positive and negative selection pressures were assessed using the SLAC, FEL and FUBAR tests available on the Datamonkey server [37].

### Structural analysis of the AC4 protein

 The amino acid sequences of the AC4 protein from all BlYSV isolates were aligned using MEGA X. The resulting alignment was manually inspected to identify nonsynonymous substitutions specific to each variant. The three-dimensional structure of the AC4 protein was predicted using AlphaFold 3 [38] under default parameters. The confidence of the models was evaluated using the predicted Local Distance Difference Test (pLDDT) scores provided by the algorithm. Regions with pLDDT values above 90 were considered high-confidence predictions, whereas regions with pLDDT values below 70 were classified as low-confidence. Structural models were visualized and edited using PyMOL v.3.0 (Schrödinger, LLC), and specific amino acid positions were highlighted for structural analysis.

### Interspecific recombination

 Sequences from nine begomoviruses showing the highest nt identity to BlYSV based on BLASTn searches were selected to construct a dataset specifically aimed at detecting interspecific recombination. This dataset comprised 37 sequences from those nine related begomoviruses (Suppl. Table S3) together with the 53 BlYSV isolates included in the other analyses. Multiple sequence alignments, obtained as previously described, were used to infer a NeighborNet network implemented in SplitsTree [39]. Recombinant events were further examined using RDP5 as previously described. Recombination analyses were conducted using both a complete dataset, consisting of 90 sequences (53 BlYSV isolates and 37 sequences from other begomoviruses), and reduced, variant-specific datasets designed to test interspecific recombination for each BlYSV variant separately. Each reduced dataset included the same 37 sequences from other begomoviruses combined with isolates belonging to a single BlYSV variant. This strategy was adopted to control for potential biases associated with large and highly heterogeneous datasets in recombination detection analyses.

## Results

A total of 119 *B. rhomboidea* and two *P. niruri* samples were collected, and 75 (73 *B. rhomboidea* and two *P. niruri*) were preliminarily positive for the presence of a begomovirus based on the detection of a 2,600-nt band after digestion of the RCA products with restriction enzymes (Suppl. Table S1). From these samples, 23 DNA-A clones (19 haplotypes) and 4 DNA-B clones (4 haplotypes) were obtained (Suppl. Table S1). BlYSV was the only species identified in this study, and the ApaI enzyme was the most efficient for cloning the DNA-A. This is the first report of this virus in Rio Grande do Norte (RN) state, and also the first time that BlYSV is found infecting *P. niruri*.

**Fig. 1:**
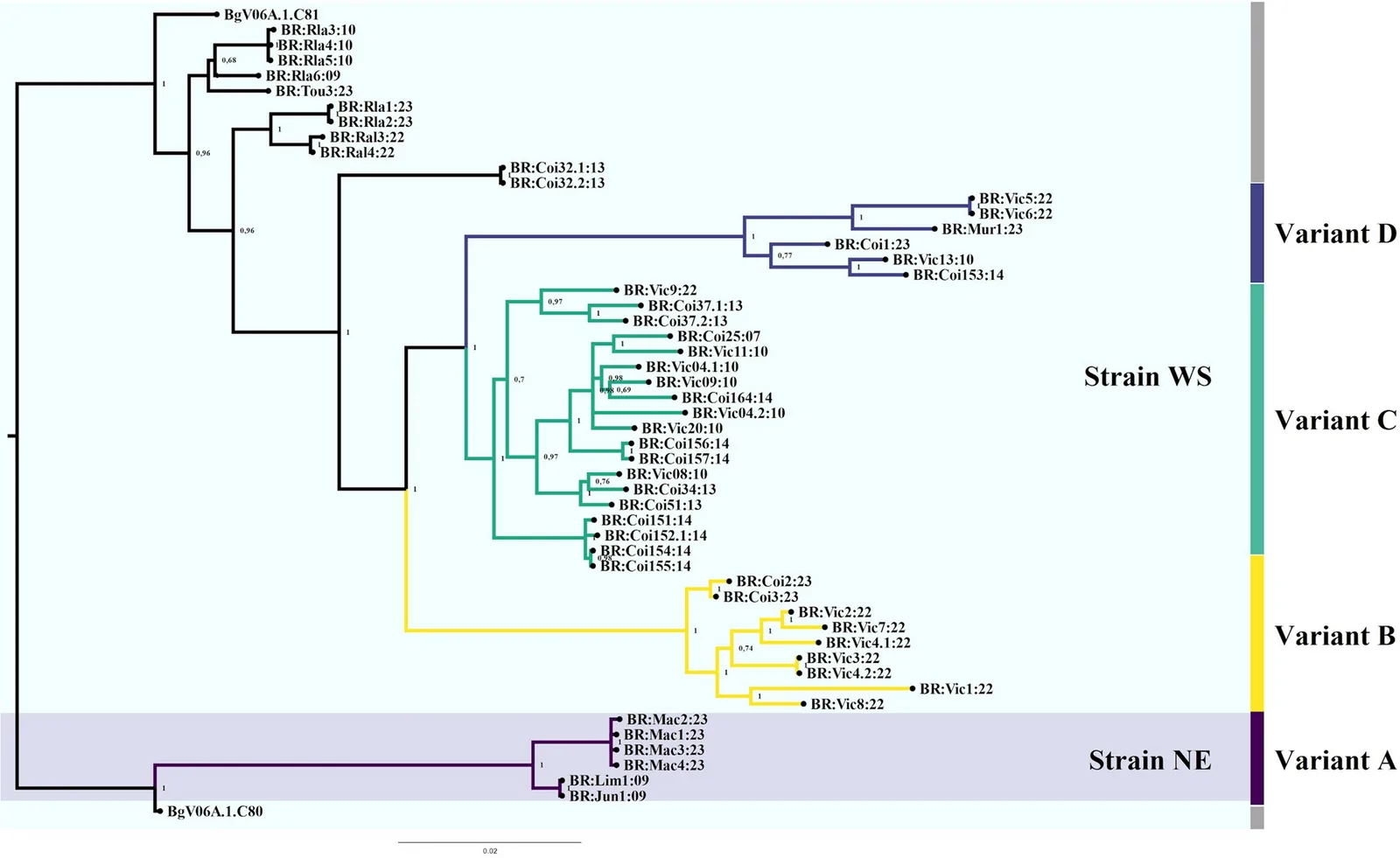
Bayesian phylogenetic tree based on the complete DNA-A sequences of Blainvillea yellow spot virus (*Begomovirus blainvilleae*; BlYSV) isolates collected over a 16-year period. Posterior probability values are indicated at the branches. Branch colors correspond to variant classification based on pairwise sequence comparisons. Isolate names include an abbreviation of the location where isolates were collected: Coi = Coimbra, Vic = Viçosa, Mac = Maceió, Jun = Junqueiro, Lim = Limoeiro, Rla = Rio Largo, Tou = Touros, Mur = Muriaé

Pairwise nucleotide sequence comparisons among the 23 DNA-A clones plus 30 additional DNA-A from GenBank revealed > 91% sequence identity amongst all the isolates (Suppl. Figure S1). Two strains, named WS (widespread) and NE (northeast), were identified. They are subdivided into four variants, named A to D, forming monophyletic clades in the DNA-A phylogenetic tree (Fig. 1) with > 97% identity between isolates within each variant (Suppl. Table S4). The prevalent WS strain includes variants B, C and D, plus a group of isolates (most from AL but also including two isolates from Coimbra, MG and the single isolate from Touros, RN) that do not form a monophyletic group and therefore were not classified as a variant. The NE strain consists of isolates from variant A found in AL.

Variant A (*n* = 6) is the most genetically distant variant (Fig. 1) and consists of isolates collected in three different municipalities of AL (Maceió, Junqueiro, and Limoeiro). Interestingly, despite their geographic proximity, no isolates from the municipality of Rio Largo were classified in this variant. Furthermore, all variant A isolates obtained in Maceió exhibited a nucleotide substitution (A2667G) within the conserved nonanucleotide motif (from 5’-TAAT*A*TTAC-3’ to 5’-TAAT*G*TTAC-3’). Sequencing of the PCR fragments from samples of isolates BR:Mac1:23 and BR:Mac2:23 confirmed this mutation. Although this region is highly conserved in the *Geminiviridae*, two other nonanucleotide motifs have been reported in members of the family. Viruses in the genera *Becurtovirus* and *Eragrovirus*, as well as one mastrevirus, have the sequence 5’-TAAGATTCC-3’ [3], and the begomoviruses Triumfetta yellow mosaic virus (*B. triumfettae*, TrYMV) and Malvaviscus yellow mosaic virus (MvYMV) carry a nanovirus-like sequence (5’-TAGTATTAC-3’) [11, 12]. No alteration in the BlYSV stem-loop structure was observed in silico (ΔG = -16.26) (Suppl. Figure S2). It is not known whether the observed mutation is also present in the cognate DNA-B components.

Variant B (*n* = 9) consists of isolates obtained in Viçosa and Coimbra (MG) collected in 2022 and 2023. All of them share a recombination event with breakpoints in the *Rep* gene and the common region, encompassing the entire *AC4* gene (Suppl. Table S5). This event is supported by the incongruence of clusters of these isolates in the *Rep* and *CP* phylogenies (Suppl. Figures S3, S4). Isolates BR:Coi157:14 (var. C) and BR:Mac1:23 (var. A) were identified as the major and minor parents, respectively, indicating an origin through “intervariant” recombination. Within this variant, a second shared recombination event was identified in BR:Vic1:22, BR:Vic2:22, BR:Vic4.1:22, and BR:Vic7:22 with breakpoints in the common region, extending until the *Trap* gene.

Variant C (*n* = 19) comprises isolates sourced from Viçosa and Coimbra (MG), spanning the years 2007 to 2022. Among the isolates sequenced in this study, the isolate BR:Vic9:22 from Viçosa was the only one classified in this variant.

**Fig. 2:**
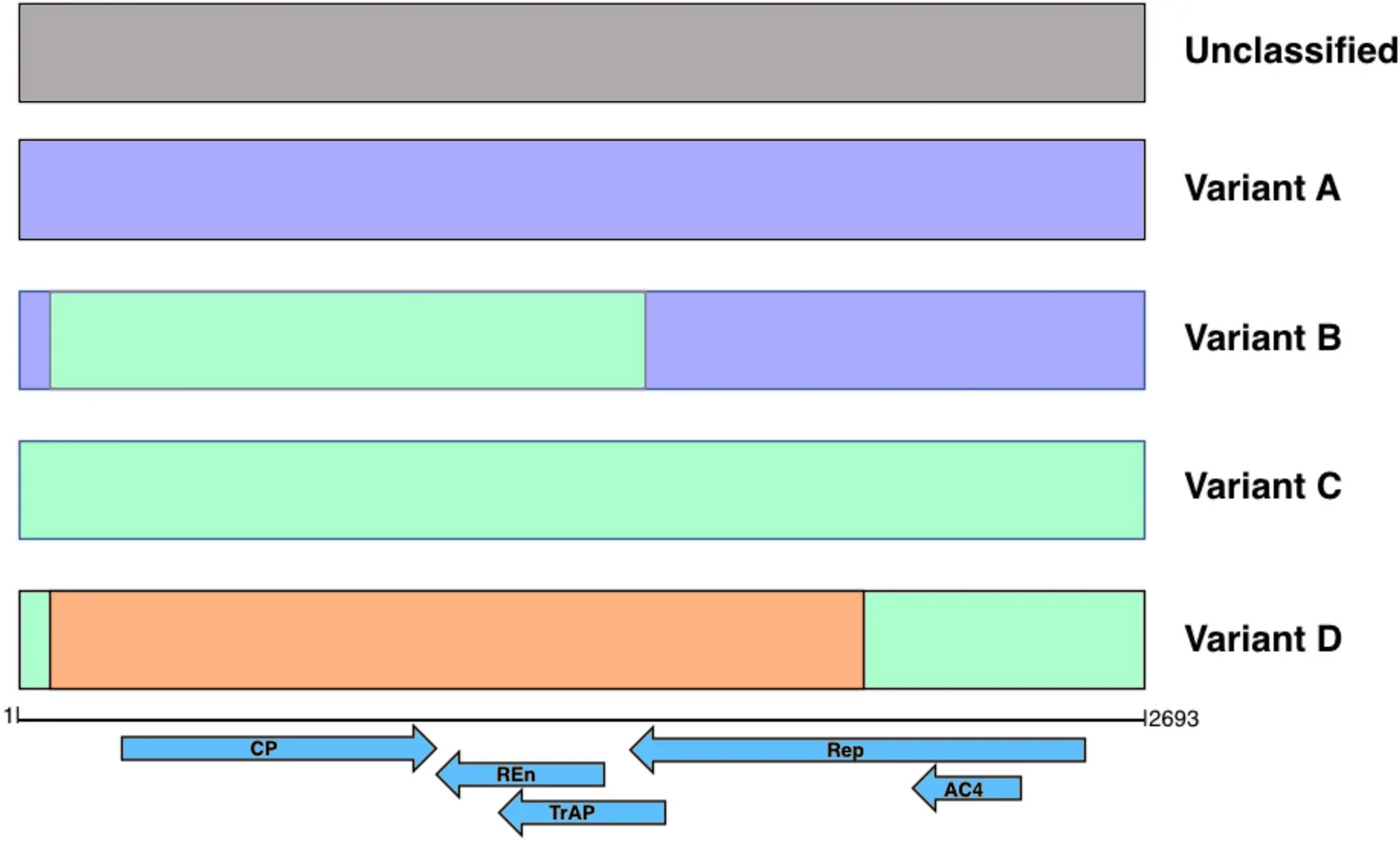
Graphical representation of the DNA-A component of Blainvillea yellow spot virus (*Begomovirus blainvilleae)* variants and the breakpoints of their intraspecific recombination events. Regions with the same color share the same parental origin

Variant D (*n* = 6) consists of isolates from the three municipalities in MG (Viçosa, Coimbra, and Muriaé). Besides having a distinct recombination event, similar to isolates from variant B, all isolates in this variant also share a second recombination event in a similar region (Suppl. Table S5). This event was previously described [15] and is also supported by incongruences between the *Rep* and *CP* trees (Suppl. Figures S3, S4). In the *Rep* phylogeny, these isolates form a distinct branch, similar to what is observed in the full DNA-A phylogeny, whereas in the *CP* phylogeny these isolates cluster together with isolates from variants B and C. A schematic representation of the DNA-A of each variant, highlighting recombination breakpoints and parental contributions, is shown in Fig. 2.

Analysis of the DNA-A phylogenetic tree (Fig. 1) reveals the formation of two major clades corresponding to the two strains. Clade I includes the vast majority of the isolates (including all isolates from MG), corresponding to the WS strain. Isolates from MG are categorized into three monophyletic subclades, corresponding to variants B, C, and D. Notably, a large number of isolates deviate from this pattern, failing to form monophyletic clades and instead displaying multiple basal lineages. This suggests an ancestral nature for these isolates relative to variants B, C, and D. This hypothesis is further corroborated by the shorter genetic distance observed between the basal group of isolates and the root.

Clade II includes six isolates from AL classified as variant A, and correspond to the NE strain. Isolates collected in Maceió in 2023 cluster together, forming a sister group with two other isolates that share a common nonanucleotide sequence. This suggests a shared origin for the mutation in their nonanucleotide. However, due to the limited sampling of this strain, more detailed inferences about the emergence of this mutation are not possible.

**Fig. 3:**
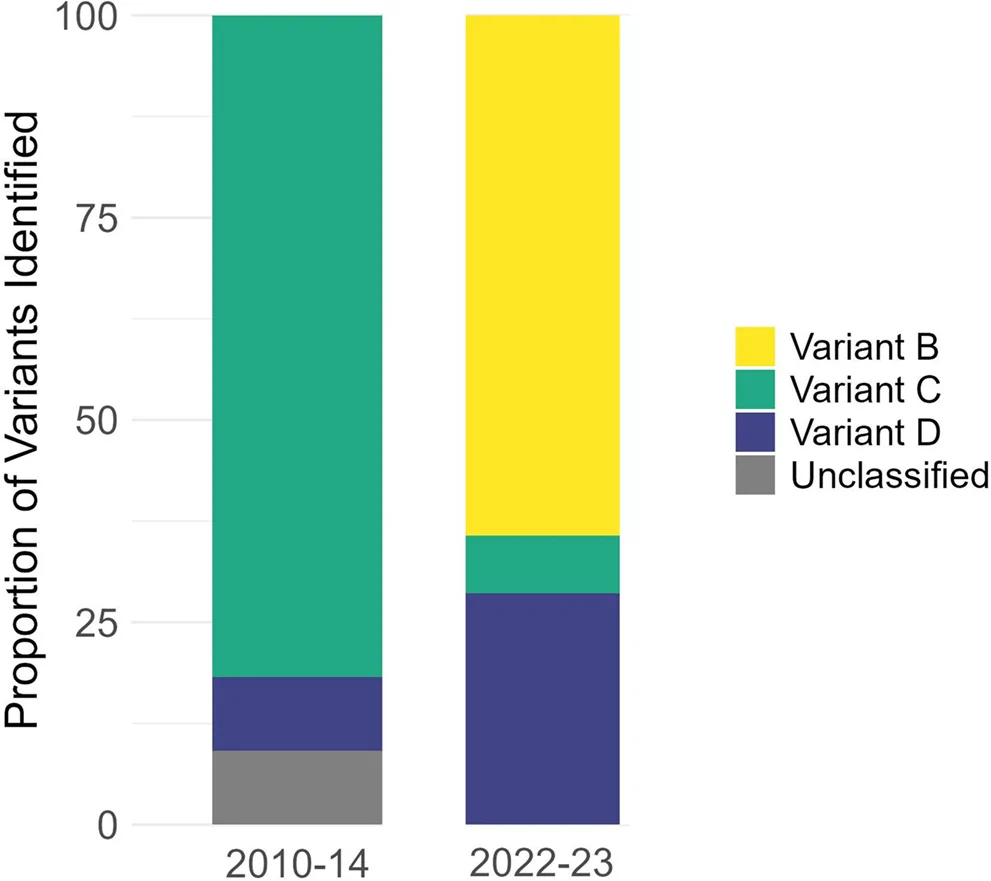
Occurrence of Blainvillea yellow spot virus (*Begomovirus blainvilleae*) variants in two time periods in the municipalities of Viçosa and Coimbra, MG

The distribution of variants collected over the years in Viçosa and Coimbra (distant less than 20 km from each other and thus treated as a single location) was analyzed in two distinct time periods (Fig. 3). Between 2010 and 2014, variant C was predominant, representing 81.8% of the sampled isolates, while variant D accounted for 9.1% of the isolates. However, a change in the distribution of the variants was observed between 2014 and 2022. During this period, variant B became predominant, accounting for 64.3% of the sampled isolates. Variant D was the second most common (28.6%) and variant C, although still present, decreased to 7.1% of the isolates. Variant occurrence over the years in other municipalities could not be analyzed due to the limited number of sequences available.

To test the hypothesis of temporal structuring among the isolates, a correlation analysis was performed between the collection date and the root-to-tip distance of the phylogenetic tree. The analysis yielded a correlation coefficient of 0.44 (R^2^ = 0.19), indicating a moderate correlation (Table 1). Besides the relatively low coefficient of determination, these values cannot be used to test for statistical significance because the data do not exhibit an independent distribution. Instead, they are partially correlated due to shared ancestry [31].

**Table 1:** Correlation analysis between the root-to-tip distance in the DNA-A phylogenetic tree and the year of sample collection of Blainvillea yellow spot virus (*Begomovirus blainvilleae*) isolates, conducted using TemPest

Dataset	*n*	Date range	Correlation coefficient	*R* ^2^	Slope (rate)	TMRCA*
Full dataset	53	16	0.44	0.19	2.731 × 10^− 3^	1994
Without variant A	47	16	0.55	0.30	2.388 × 10^− 3^	1995

*TMRCA: time to most recent common ancestor

Correlation analysis was then conducted excluding variant A isolates, which were identified as outliers based on the TempEst graph. These isolates could be evolving at a different evolutionary rate, but this could be masked due to undersampling. Excluding variant A isolates increased the correlation coefficient to 0.55 (R² = 0.30) (Table 1), indicating that the population, at least for the WS strain, is partially structured over time. The X-axis intercept indicates the time for the most recent common ancestor (TMRCA) to be around 1995, shortly before the virus was first reported [40].

**Fig. 4:**
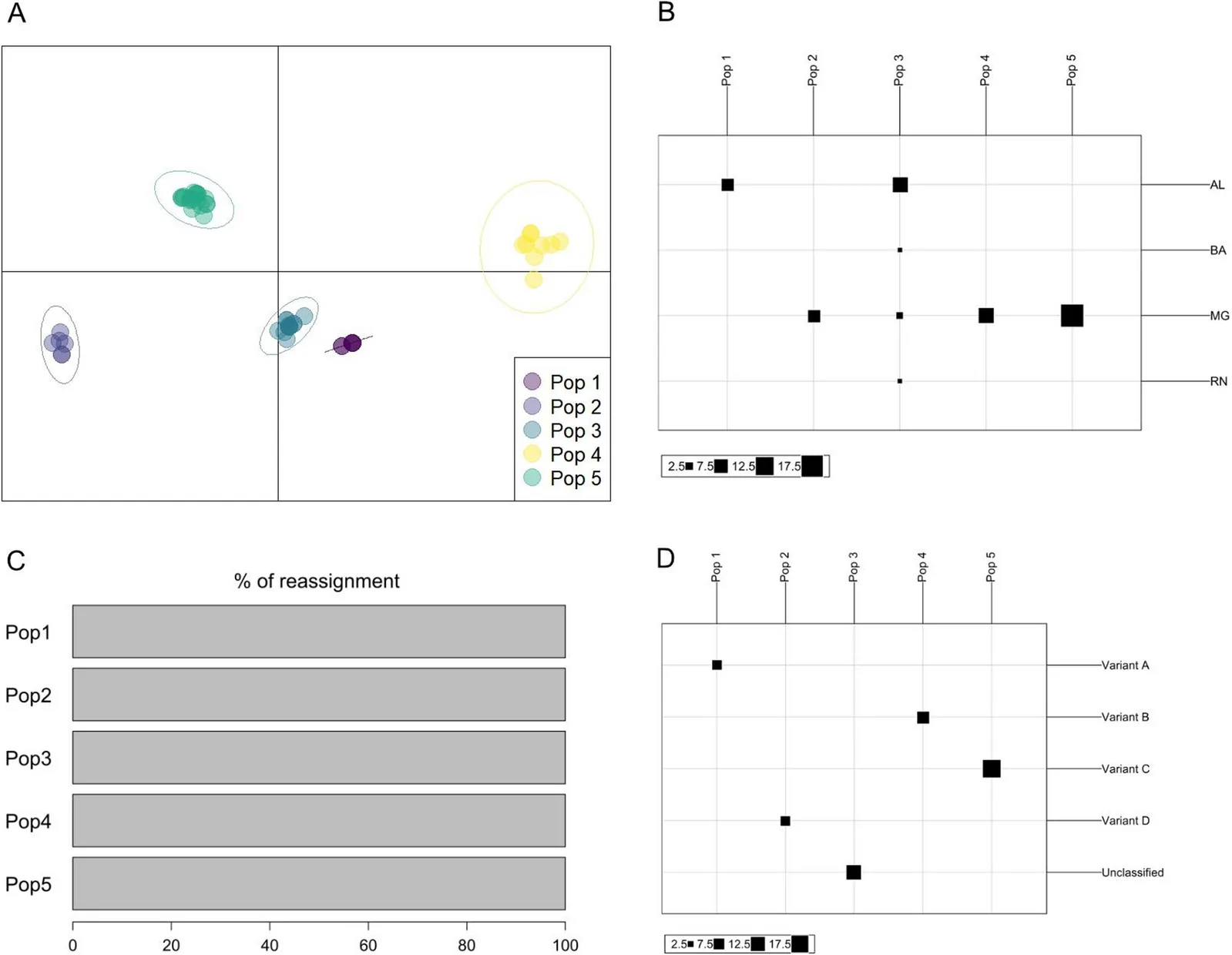
Multivariate statistical clustering analysis of population subdivision using Discriminant Analysis of Principal Components (DAPC) for Blainvillea yellow spot virus (*Begomovirus blainvilleae*). (**A)** DAPC scatter plot indicating population subdivision. (**B)** Comparison between groups inferred by *k*-stat (columns) and isolate origin (rows). (**C)** Percentage of successful reassignment after randomization retaining six principal components. (**D)** Comparison between groups inferred by *k*-stat (columns) and variants classification (rows)

Based on six principal components, DAPC confirmed the division of the BlYSV population into five subpopulations (*k* = 5), consistent with the variant classification (Fig. 4). The isolates that were not classified into a variant cluster within a single subpopulation (pop3) with high membership probabilities. Reassignment percentages indicate that DAPC is effective in separating the groups, with all individuals correctly assigned to their original groups. The high differentiation between subpopulations is supported by the genetic variability observed within each group relative to the total variability, indicated by the Nst index ranging from 0.51 to 0.85 (Table 2).

**Table 2:** Results of subdivision test (Nst) performed among the five subpopulations* of Blainvillea yellow spot virus (*Begomovirus blainvilleae*) as determined by Discriminant Analysis of Principal Components (DAPC)

Subpopulations		Nst^#^
pop1	pop2	0.81
pop1	pop3	0.79
pop1	pop4	0.86
pop1	pop5	0.84
pop2	pop3	0.59
pop2	pop4	0.72
pop2	pop5	0.59
pop3	pop4	0.67
pop3	pop5	0.51
pop4	pop5	0.66

^*^pop1 corresponds to variant A, pop2 to variant D, pop4 to variant B, and pop5 to variant C. pop3 isolates were not classified into a variant

^#^Values from 0 to 0.05 indicate little genetic differentiation between subpopulations; 0.05 to 0.15, moderate differentiation; 0.15 to 0.25, great differentiation; >0.25 high differentiation

Overall, the BlYSV population exhibited a nucleotide diversity (π) value of 0.049, although at least half of this genetic variability can be attributed to differences among variants (Table 3). The difference of nucleotide diversity between variant B and C isolates is not statistically significant (Suppl. Figure S5), while variant D and subpopulation 3 exhibited the highest nucleotide diversity (π = 0.025). Intriguingly, variant A isolates exhibit a much lower nucleotide diversity (π = 0.007) than the other subpopulations.

**Table 3:** Genetic variability indices for Blainvillea yellow spot virus (*Begomovirus blainvilleae*) population and its five subpopulations* as determined by Discriminant Analysis of Principal Components (DAPC)

Subpopulation	*n* ^#^	π	h	Hd	ϴ	s	Eta	k
pop1	6	0.007	5	0.93	0.00574	35	35	17.7
pop2	6	0.025	5	0.93	0.02287	136	139	65.9
pop3	13	0.025	10	0.96	0.02832	223	234	65.8
pop4	9	0.016	8	0.97	0.01913	132	138	43.1
pop5	19	0.019	18	0.99	0.02480	218	231	49.7
TOTAL	53	0.049	46	0.99	0.05806	579	695	129.0

^*^pop1 corresponds to variant *A,* pop2 to variant *D,* pop4 to variant *B,* and pop5 to variant *C*. pop3 isolates were not classified into a variant

^**#**^*n,* number of sequences; π, nucleotide diversity; *h,* haplotype number; *Hd,* haplotype diversity; *ϴ,* mutation rate, *Eta,* total number of mutations; *s,* total number of polymorphic sites; *k,* average number of nucleotide differences

Values obtained in Tajima’s D, Fu and Li’s D* and F* tests did not differ statistically from zero in any protein × subpopulation analysis, indicating neutral equilibrium across the entire BlYSV population (Suppl. Table S6). dN/dS values indicated that the AC4 protein of variant B isolates is under positive or diversifying selection. All other analyzed proteins showed negative selection (dN/dS < 1). The CP exhibited the strongest negative selection pressure (dN/dS ranging from 0.0098 to 0.0847). Consistent with the negative selection pressure observed in the genes, most amino acid sites were under negative selection in all three tests (Suppl. Table S7). A few sites under positive selection were observed in Rep, REn, TrAP and AC4. Analysis of variant B isolates did not identify sites under positive selection in AC4.

**Fig. 5:**
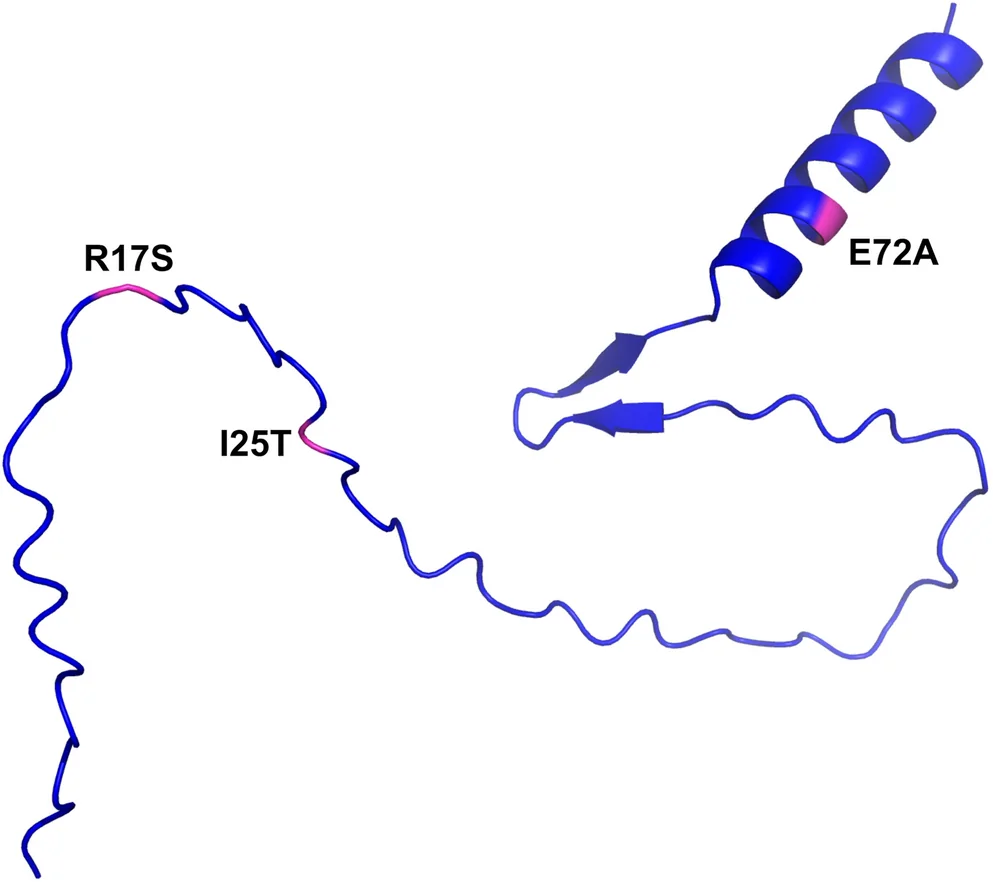
Three-dimensional structure of the AC4 protein of Blainvillea yellow spot virus (*Begomovirus blainvilleae*) isolate BR:Vic4.1:22 (variant B). Amino acid residues highlighted in pink indicate substitutions that are exclusive to this variant. Single-letter codes correspond to the following amino acids: R, arginine; S, serine; I, isoleucine; T, threonine; E, glutamic acid (glutamate); A, alanine

Analysis of the amino acid alignment of the AC4 protein revealed three nonsynonymous substitutions (R17S, I25T, and E72A) that are exclusive to variant B and absent in all other isolates. Structural predictions generated using AlphaFold 3 (Fig. 5) indicate that the AC4 protein in variant B isolates adopts a globally weakly compact conformation similar to all other BlYSV isolates, with no clear structural change. The E72A mutation is located within a well-defined α-helical element in the C-terminal region of AC4, which is predicted with very high confidence (pLDDT > 90). In contrast, the remaining two mutations, R17S and I25T, are positioned in the N-terminal region, which is predominantly disordered and characterized by lower confidence scores (pLDDT > 70), consistent with intrinsically disordered regions (IDR).

NeighborNet network analysis revealed the formation of a well-defined cluster, with all BlYSV isolates grouping together (Suppl. Figure S6). Tomato leaf curl purple vein virus (*B. solanumviolavenae*, ToLCPVV) and Macroptilium yellow spot virus (*B. macroptilimaculae*, MacYSV) were recovered as the closest external taxa; however, BlYSV remained genetically cohesive and monophyletic, with no strong reticulations crossing the cluster. Interspecific recombination analysis performed in RDP5 did not detect any recombination events, either when the complete dataset was analyzed (53 BlYSV isolates), or when each variant (A–D) was evaluated separately (Suppl. Table S8). An exception was observed in the dataset composed of isolates that did not form phylogenetically distinct variants (pop3), in which a recombination event was detected involving BlYSV and shared with a subset of MacYSV isolates (4 out of 6 isolates analyzed). However, parental assignment for this genomic region was inconsistent, as BlYSV, MacYSV, and ToLCPVV alternated between being inferred as the recombinant or the minor parent in ToLCPVV isolates and in the same four MacYSV isolates, depending on the dataset analyzed.

## Discussion

Following the initial detection of *Bemisia tabaci* MEAM1 in Brazil in the mid-1990’s, numerous reports of begomoviruses causing significant economic losses in cultivated plants, particularly tomatoes, began to surface [41]. A high begomovirus diversity infecting this crop was observed, and the indigenous origin of the viruses (from non-cultivated plant hosts) became rapidly evident. A comprehensive study carried out about ten years after the emergence of the new viruses reported that the begomovirus populations in tomatoes and associated weed hosts evolved at rapid substitution rates, were highly recombinant, and geographically structured [13].

BlYSV was first detected in 1999 [40], only a few years after the estimated time to the most recent common ancestor (TMRCA), and so far has only been reported in Brazil. Notably, it is the only begomovirus reported infecting *B. rhomboidea*. Although reports of this virus infecting other hosts are rare [13], we have found it in the non-cultivated plant *Phyllanthus niruri*. Thus, BlYSV in *B. rhomboidea* can be considered a “sealed container” pathosystem, as defined by García-Arenal & Zerbini [2].

Despite its narrow host range, BlYSV exhibits relatively high nucleotide diversity compared to other species infecting cultivated plants such as BGMV, tomato common mosaic virus (*B. solanumvulgarismusivi*, ToCmMV), tomato chlorotic mottle virus (*B. solanumpallidivariati*, ToCMoV), tomato severe rugose virus (*B. solanumseverugosi*, ToSRV) and tomato yellow vein streak virus (*B. solanumflavusvenae*, ToYVSV) [13, 15]. Most of this variability can be attributed to its subdivision into five subpopulations, and is comparable to that observed in subpopulations of Macroptilium yellow spot virus (*B. macroptilimaculae*, MacYSV) [15]. Our data demonstrate the coexistence of two distinct strains. The WS strain is widely distributed in the northeast and southeast regions of the country and is subdivided into three variants (B, C and D) plus a group of unclassified isolates. Meanwhile, the NE strain has only been found so far in the northeast region (Alagoas and Pernambuco states) [42]. Phylogenetic analysis suggests that the northeast region is likely the center of origin for the WS strain, which later migrated to the southeast region.

Although interspecific recombination among begomoviruses was detected in a dataset comprising only strain WS isolates that are not classified into variants (pop3 in the DAPC analysis), the combined evidence supports the genetic cohesiveness and monophyly of BlYSV. Importantly, we believe that the detected recombination signal predates the diversification of BlYSV variants, with its phylogenetic footprint becoming progressively eroded over time, likely due to the subsequent accumulation of mutations and extensive intraspecific recombination. Consequently, this ancestral signal may only be detectable in pop3 isolates, which likely represent lineages closer to the ancestral state. The inconsistency in parental assignment for the recombinant region may reflect methodological limitations inherent to recombination detection algorithms, or alternatively, a complex shared evolutionary history among these species, which belong to the same phylogenetic clade [43]. This warrants further investigation.

Variant C isolates predominated in the municipalities of Viçosa and Coimbra during the period spanning 2010 to 2014 and were already present at least three years earlier, as evidenced by isolate BR:Coi25:07, the first sequenced BlYSV genome. However, after recombining with a NE strain (variant A) isolate, variant C gave rise to variant B, which quickly replaced it.

Although negative selection is the most influential evolutionary force acting on begomovirus populations [15, 44], positive selection was found to be acting on the *AC4* gene of variant B and pop3 isolates. The fact that *AC4* is often under positive selection has been reported before and is thought to be correlated with the functions of this protein as a pathogenicity factor [45, 46]. Following the recombination event that originated variant B isolates, AC4 mutations that enable evasion of the plant immune system may be associated with its increased prevalence in Viçosa and Coimbra. They can also be linked to the *P. niruri* spillover event observed in pop3. Indeed, three amino acid substitutions exclusive to variant B isolates were identified in the AC4 protein (R17S, I25T, and E72A). Notably, these substitutions are absent from the closest putative parental sequence of the recombinant region, suggesting their rapid fixation following the recombination event. Two of these mutations are located within an intrinsically disordered region (IDR) of AC4. Intrinsically disordered regions are commonly associated with protein–protein interactions and functional plasticity. Nevertheless, although amino acid substitutions within IDRs may alter AC4 function without imposing strong structural constraints [45, 46], additional studies are required to determine whether these three sites contribute to a potential increase in fitness of variant B isolates.

BlYSV DNA-A shows extensive intraspecific recombination. About 1/3 of the isolates evaluated in this dataset showed recombination events (18 out of 53). Similar results were reported for the DNA-B [15]. Begomovirus populations are often recombinant and this affects various aspects of their evolution. Despite this mechanism being crucial for increasing viral diversity, it may also serve to eliminate deleterious mutations. The intergenic region and the *Rep* gene are important recombination hotspots, thus exhibiting relatively higher genetic variability.

Although it does not encode any proteins, the intergenic region in begomoviruses plays a crucial role in the viral life cycle [8]. This region is highly variable among different species but exhibits high identity among isolates of the same species. It contains a CG-rich region of approximately 30 nt that is common to both segments of bisegmented begomoviruses and forms a hairpin-shaped secondary structure with a conserved nonanucleotide motif in the loop, serving as a replication origin [47]. Isolates collected in different plants obtained in Maceió in 2023 have a mutation in the nonanucleotide motif that does not alter the cleavage site of the Rep protein or the secondary structure of the hairpin. This mutation is not found in isolates of the same variant obtained in nearby municipalities in the year 2009. However, the same mutation is present in a phylogenetically related isolate from Pernambuco obtained in 2021 (approximately 231 km from Maceió), suggesting that it is not due to a sporadic event [42]. The biological effect of this mutation is unknown. Nevertheless, it deserves attention because it is in an extremely conserved region with high potential for recombination.

Two hypotheses for the emergence and spread of this mutation are proposed. The first hypothesis suggests that the effect is neutral, and its spread occurs through genetic drift. The low genetic variability observed in this subpopulation supports this hypothesis and suggests genetic bottlenecks that amplify the impacts of genetic drift. However, a second hypothesis suggesting an increase in viral fitness, justifying the spread of this mutation across different states due to natural selection, cannot be ruled out. Therefore, it is important to determine the biological effects of this alternative nonanucleotide, as well as monitor the evolutionary dynamics of this strain in the northeast region. This can contribute to a better understanding of how viruses from the family *Geminiviridae* restructure their origin of replication.

We have carried out a detailed analysis of the genetic variability and population structure of BlYSV, a virus which is widespread in the non-cultivated host *Blainvillea rhomboidea*, but is mostly restricted to this host. Our results confirm the high genetic variability of this virus which was hinted at in previous studies, with the isolates being subdivided into two strains and four variants. One of the variants seems to have enhanced fitness due to a recombination event followed by positive selection in the *AC4* gene, while another variant has a mutation in the extremely conserved origin of replication. Our future studies will be directed at understanding the impact of these mutations in viral fitness, and whether this could facilitate spillover of BlYSV to other hosts, including cultivated plants.

## Supplementary information

Below is the link to the electronic supplementary material.

Supplementary Material 1

## Data Availability

The nucleotide sequences generated in this study are available in GenBank under accession numbers PQ602568–PQ602590 (DNA-A) and PQ619930–PQ619933 (DNA-B).
