# Supplementary material for: Genetic variability of blainvillea yellow spot virus (Begomovirus blainvilleae) reveals recombinant variants and a distinct nonanucleotide motif
Source: Arch Virol. 2026 Jul 20;171(8):231. doi: 10.1007/s00705-026-06689-z (PMC13385248; doi:10.1007/s00705-026-06689-z)
Supplement: Supplementary file 1 — Supplementary Material 1 [file 705_2026_6689_MOESM1_ESM.docx]

**SUPPLEMENTARY MATERIAL**

**Suppl. Table S1.** Blainvillea yellow spot virus (*Begomovirus blainvilleae*) isolates/clones obtained in this work.

| **Isolate** | **GenBank access number** | **Component** | **Host** | **Location^1^** | **Date of collection** | **Enzyme** |
| --- | --- | --- | --- | --- | --- | --- |
| BR:Vic1:22 | PQ602568 | DNA-A | *Blainvillea rhomboidea* | Viçosa, MG | May, 2022 | *ApaI* |
| BR:Vic2:22 | PQ602569 | DNA-A | *Blainvillea rhomboidea* | Viçosa, MG | May, 2022 | *ApaI* |
| BR:Vic3:22 | PQ602570 | DNA-A | *Blainvillea rhomboidea* | Viçosa, MG | May, 2022 | *ApaI* |
| BR:Vic4.1:22 | PQ602571 | DNA-A | *Blainvillea rhomboidea* | Viçosa, MG | May, 2022 | *ApaI* |
| BR:Vic4.2:22 | PQ602572 | DNA-A | *Blainvillea rhomboidea* | Viçosa, MG | May, 2022 | *ApaI* |
| BR:Vic5:22 | PQ602573 | DNA-A | *Blainvillea rhomboidea* | Viçosa, MG | May, 2022 | *ApaI* |
|  | PQ619933 | DNA-B |  |  |  | *XbaI* |
| BR:Vic6:22 | PQ602574 | DNA-A | *Blainvillea rhomboidea* | Viçosa, MG | May, 2022 | *ApaI* |
| BR:Vic7:22 | PQ602575 | DNA-A | *Blainvillea rhomboidea* | Viçosa, MG | May, 2022 | *ApaI* |
| BR:Vic8:22 | PQ602576 | DNA-A | *Blainvillea rhomboidea* | Viçosa, MG | May, 2022 | *ApaI* |
| BR:Vic9:22 | PQ602577 | DNA-A | *Blainvillea rhomboidea* | Viçosa, MG | May, 2022 | *ApaI* |
| BR:Mur1:23 | PQ602578 | DNA-A | *Blainvillea rhomboidea* | Muriaé, MG | Jan, 2023 | *XbaI* |
| BR:Coi1:23 | PQ602579 | DNA-A | *Blainvillea rhomboidea* | Coimbra, MG | May, 2023 | *ApaI* |
| BR:Coi2:23 | PQ602580 | DNA-A | *Blainvillea rhomboidea* | Coimbra, MG | May, 2023 | *ApaI* |
| BR:Coi3:23 | PQ602581 | DNA-A | *Blainvillea rhomboidea* | Coimbra, MG | May, 2023 | *ApaI* |
| BR:Mac1:23 | PQ602582 | DNA-A | *Blainvillea rhomboidea* | Maceió, AL | May, 2023 | *ApaI* |
| BR:Mac2:23 | PQ602583 | DNA-A | *Blainvillea rhomboidea* | Maceió, AL | May, 2023 | *ApaI* |
| BR:Mac3:23 | PQ602584 | DNA-A | *Blainvillea rhomboidea* | Maceió, AL | May, 2023 | *ApaI* |
| BR:Mac4:23 | PQ602585 | DNA-A | *Blainvillea rhomboidea* | Maceió, AL | May, 2023 | *ApaI* |
| BR:Rla1:23 | PQ602586 | DNA-A | *Blainvillea rhomboidea* | Rio Largo, AL | May, 2023 | *PstI* |
| BR:Rla2:23 | PQ602587 | DNA-A | *Blainvillea rhomboidea* | Rio Largo, AL | May, 2023 | *PstI* |
|  | PQ619932 | DNA-B |  |  |  | *PstI* |
| BR:Rla3:20 | PQ602588 | DNA-A | *Phyllanthus niruri* | Rio Largo, AL | Aug, 2020 | *ApaI* |
| BR:Rla4:20 | PQ602589 | DNA-A | *Phyllanthus niruri* | Rio Largo, AL | Aug, 2020 | *HindIII* |
| BR:Tou1:23 | PQ619931 | DNA-B | *Blainvillea rhomboidea* | Touros, RN | May, 2023 | *XbaI* |
| BR:Tou3:23 | PQ602590 | DNA-A | *Blainvillea rhomboidea* | Touros, RN | May, 2023 | *ApaI* |
| BR:Tou4:23 | PQ619930 | DNA-B | *Blainvillea rhomboidea* | Touros, RN | May, 2023 | *XbaI* |

^1^State abbreviations: AL, Alagoas; MG, Minas Gerais; RN, Rio Grande do Norte

**Suppl. Table S2.** DNA-A sequences of Blainvillea yellow spot virus (*Begomovirus blainvilleae*) available in GenBank and used in this work.

| **Isolate** | **GenBank access number** | **City** | **State** | **Year of collection** | **Host** |
| --- | --- | --- | --- | --- | --- |
| BR:Coi164:14 | MT626996 | Coimbra | Minas Gerais | 2014 | *Blainvillea rhomboidea* |
| BR:Coi157:14 | MT626995 | Coimbra | Minas Gerais | 2014 | *Blainvillea rhomboidea* |
| BR:Coi156:14 | MT626994 | Coimbra | Minas Gerais | 2014 | *Blainvillea rhomboidea* |
| BR:Coi155:14 | MT626993 | Coimbra | Minas Gerais | 2014 | *Blainvillea rhomboidea* |
| BR:Coi154:14 | MT626992 | Coimbra | Minas Gerais | 2014 | *Blainvillea rhomboidea* |
| BR:Coi153:14 | MT626991 | Coimbra | Minas Gerais | 2014 | *Blainvillea rhomboidea* |
| BR:Coi152.1:14 | MT626990 | Coimbra | Minas Gerais | 2014 | *Blainvillea rhomboidea* |
| BR:Coi151:14 | MT626989 | Coimbra | Minas Gerais | 2014 | *Blainvillea rhomboidea* |
| BR:Coi51:13 | MT626988 | Coimbra | Minas Gerais | 2013 | *Blainvillea rhomboidea* |
| BR:Coi37.2:13 | MT626987 | Coimbra | Minas Gerais | 2013 | *Blainvillea rhomboidea* |
| BR:Coi37.1:13 | MT626986 | Coimbra | Minas Gerais | 2013 | *Blainvillea rhomboidea* |
| BR:Coi34:13 | MT626985 | Coimbra | Minas Gerais | 2013 | *Blainvillea rhomboidea* |
| BR:Coi32.2:13 | MT626984 | Coimbra | Minas Gerais | 2013 | *Blainvillea rhomboidea* |
| BR:Coi32.1:13 | MT626983 | Coimbra | Minas Gerais | 2013 | *Blainvillea rhomboidea* |
| BR:Vic20:10 | KC706522 | Viçosa | Minas Gerais | 2010 | *Physalis* sp. |
| BR:Vic13:10 | KC706521 | Viçosa | Minas Gerais | 2010 | *Blainvillea rhomboidea* |
| BR:Vic11:10 | KC706520 | Viçosa | Minas Gerais | 2010 | *Blainvillea rhomboidea* |
| BR:Vic09:10 | KC706519 | Viçosa | Minas Gerais | 2010 | *Blainvillea rhomboidea* |
| BR:Vic08:10 | KC706518 | Viçosa | Minas Gerais | 2010 | *Blainvillea rhomboidea* |
| BR:Vic04.2:10 | KC706517 | Viçosa | Minas Gerais | 2010 | *Blainvillea rhomboidea* |
| BR:Vic04.1:10 | KC706516 | Viçosa | Minas Gerais | 2010 | *Blainvillea rhomboidea* |
| BR:Jun1:09 | JX871394 | Junqueiro | Alagoas | 2009 | *Blainvillea rhomboidea* |
| BR:Lim1:09 | JX871393 | Limoeiro | Alagoas | 2009 | *Blainvillea rhomboidea* |
| BR:Rla6:09 | JX871392 | Rio Largo | Alagoas | 2009 | *Blainvillea rhomboidea* |
| BR:Rla5:10 | JX871391 | Rio Largo | Alagoas | 2010 | *Blainvillea rhomboidea* |
| BR:Rla4:10 | JX871390 | Rio Largo | Alagoas | 2010 | *Blainvillea rhomboidea* |
| BR:Rla3:10 | JX871389 | Rio Largo | Alagoas | 2010 | *Blainvillea rhomboidea* |
| BgV06A.1.C81 | JF694476 | Rio Largo | Alagoas | 2009/10 | *Blainvillea rhomboidea* |
| BgV06A.1.C80 | JF694468 | - | Bahia | 2009/10 | *Blainvillea rhomboidea* |
| BR:Coi25:07 | EU710756 | Coimbra | Minas Gerais | 2007 | *Blainvillea rhomboidea* |

**Suppl. Table S3.** List of begomovirus species/isolates used in the interspecific recombination analysis.

| **GenBank access number** | **Common name** | **Acronym** | **SpeciesScientific name** |
| --- | --- | --- | --- |
| MN508222 | Sida golden mosaic Brazil virus | SiGMBRV | *Begomovirus sidaureibrazilense* |
| FN436001 | Sida golden mosaic Brazil virus | SiGMBRV | *B. sidaureibrazilense* |
| KY196217 | tomato leaf curl purple vein virus | ToLCPVV | *B. solanumviolavenae* |
| KY196216 | tomato leaf curl purple vein virus | ToLCPVV | *B. solanumviolavenae* |
| KY196221 | tomato leaf curl purple vein virus | ToLCPVV | *B. solanumviolavenae* |
| KJ939737 | bean golden mosaic virus | BGMV | *B. costai* |
| KJ939756 | bean golden mosaic virus | BGMV | *B. costai* |
| KJ939772 | bean golden mosaic virus | BGMV | *B. costai* |
| KJ939783 | bean golden mosaic virus | BGMV | *B. costai* |
| KJ939795 | bean golden mosaic virus | BGMV | *B. costai* |
| KX691398 | Macroptilium yellow spot virus | MacYSV | *B. macroptilimaculae* |
| JN419013 | Macroptilium yellow spot virus | MacYSV | *B. macroptilimaculae* |
| KC004111 | Macroptilium yellow spot virus | MacYSV | *B. macroptilimaculae* |
| KC004121 | Macroptilium yellow spot virus | MacYSV | *B. macroptilimaculae* |
| KJ939861 | Macroptilium yellow spot virus | MacYSV | *B. macroptilimaculae* |
| KT779561 | Macroptilium yellow spot virus | MacYSV | *B. macroptilimaculae* |
| JF803253 | tomato interveinal chlorosis virus | ToICV | *B. solanumintervenae* |
| PP639095 | tomato interveinal chlorosis virus | ToICV | *B. solanumintervenae* |
| PP639094 | tomato interveinal chlorosis virus | ToICV | *B. solanumintervenae* |
| PP639093 | tomato interveinal chlorosis virus | ToICV | *B. solanumintervenae* |
| KX691397 | Macroptilium common mosaic virus | MacCMV | *B. macroptilicommunis* |
| KX691396 | Macroptilium common mosaic virus | MacCMV | *B. macroptilicommunis* |
| PQ261152 | Macroptilium yellow vein virus | MacYVV | *B. macroptilivenae* |
| PQ261149 | Macroptilium yellow vein virus | MacYVV | *B. macroptilivenae* |
| PQ261147 | Macroptilium yellow vein virus | MacYVV | *B. macroptilivenae* |
| PQ261140 | Macroptilium yellow vein virus | MacYVV | *B. macroptilivenae* |
| PQ261132 | Macroptilium yellow vein virus | MacYVV | *B. macroptilivenae* |
| MW573999 | tomato severe rugose virus | ToSRV | *B. solanumseverugosi* |
| MT627050 | tomato severe rugose virus | ToSRV | *B. solanumseverugosi* |
| MT627071 | tomato severe rugose virus | ToSRV | *B. solanumseverugosi* |
| KC004089 | tomato severe rugose virus | ToSRV | *B. solanumseverugosi* |
| MW573988 | tomato severe rugose virus | ToSRV | *B. solanumseverugosi* |
| KC706561 | tomato chlorotic mottle virus | ToCMoV | *B. solanumpallidivariati* |
| KC706541 | tomato chlorotic mottle virus | ToCMoV | *B. solanumpallidivariati* |
| AF490004 | tomato chlorotic mottle virus | ToCMoV | *B. solanumpallidivariati* |
| MT733804 | tomato chlorotic mottle virus | ToCMoV | *B. solanumpallidivariati* |
| MT215003 | tomato chlorotic mottle virus | ToCMoV | *B. solanumpallidivariati* |

**Suppl. Table S4.** Classification of Blainvillea yellow spot virus (*Begomovirus blainvilleae*) isolates according to strains, variants and subpopulations.

| **Isolate** | **Strain^*^** | **Variant^#^** | **Subpopulation^$^** |
| --- | --- | --- | --- |
| BR:Vic1:22 | WS | B | Pop4 |
| BR:Vic2:22 | WS | B | Pop4 |
| BR:Vic3:22 | WS | B | Pop4 |
| BR:Vic4.1:22 | WS | B | Pop4 |
| BR:Vic4.2:22 | WS | B | Pop4 |
| BR:Vic5:22 | WS | D | Pop2 |
| BR:Vic6:22 | WS | D | Pop2 |
| BR:Vic7:22 | WS | B | Pop4 |
| BR:Vic8:22 | WS | B | Pop4 |
| BR:Vic9:22 | WS | C | Pop5 |
| BR:Mur1:23 | WS | D | Pop2 |
| BR:Coi1:23 | WS | D | Pop2 |
| BR:Coi2:23 | WS | B | Pop4 |
| BR:Coi3:23 | WS | B | Pop4 |
| BgV06A.1.C80 | WS | -^&^ | Pop3 |
| BgV06A.1.C81 | WS | - | Pop3 |
| BR:Mac1:23 | NE | A | Pop1 |
| BR:Mac2:23 | NE | A | Pop1 |
| BR:Mac3:23 | NE | A | Pop1 |
| BR:Mac4:23 | NE | A | Pop1 |
| BR:Coi151:14 | WS | C | Pop5 |
| BR:Coi152.1:14 | WS | C | Pop5 |
| BR:Coi153:14 | WS | D | Pop2 |
| BR:Coi154:14 | WS | C | Pop5 |
| BR:Coi155:14 | WS | C | Pop5 |
| BR:Coi156:14 | WS | C | Pop5 |
| BR:Coi157:14 | WS | C | Pop5 |
| BR:Coi164:14 | WS | C | Pop5 |
| BR:Coi25:07 | WS | C | Pop5 |
| BR:Coi32.1:13 | WS | - | Pop3 |
| BR:Coi32.2:13 | WS | - | Pop3 |
| BR:Coi34:13 | WS | C | Pop5 |
| BR:Coi37.1:13 | WS | C | Pop5 |
| BR:Coi37.2:13 | WS | C | Pop5 |
| BR:Coi51:13 | WS | C | Pop5 |
| BR:Jun1:09 | NE | A | Pop1 |
| BR:Lim1:09 | NE | A | Pop1 |
| BR:Rla3:10 | WS | - | Pop3 |
| BR:Rla4:10 | WS | - | Pop3 |
| BR:Rla5:10 | WS | - | Pop3 |
| BR:Rla6:09 | WS | - | Pop3 |
| BR:Vic04.1:10 | WS | C | Pop5 |
| BR:Vic04.2:10 | WS | C | Pop5 |
| BR:Vic08:10 | WS | C | Pop5 |
| BR:Vic09:10 | WS | C | Pop5 |
| BR:Vic11:10 | WS | C | Pop5 |
| BR:Vic13:10 | WS | D | Pop2 |
| BR:Vic20:10 | WS | C | Pop5 |
| BR:Rla1:23 | WS | - | Pop3 |
| BR:Rla2:23 | WS | - | Pop3 |
| BR:Ral3:20 | WS | - | Pop3 |
| BR:Ral4:20 | WS | - | Pop3 |
| BR:Tou3:23 | WS | - | Pop3 |

^*^According to the criterion of >94% nucleotide sequence identity for the DNA-A {Brown, 2015 #19477}

^#^According to the criteria of >96% nucleotide sequence identity for the DNA-A and forming a monophyletic cluster in the phylogenetic tree

^$^According to Discriminant Analysis of Principal Components (DAPC)

^&^Not classified into a variant

**Suppl. Table S5.** Intraspecific recombination events detected in Blainvillea yellow spot virus (*Begomovirus blainvilleae*) DNA-A.

| **Event** | **Recombinant isolates** | **Recombination  breakpoints**^*^ | | **Parents** | | **Method^#^** | ***p*-value**^&^ |  |
| --- | --- | --- | --- | --- | --- | --- | --- | --- |
|  |  |  |  |  |  |  |  |  |
|  |  | **Begin** | **End** | **Major** | **Minor** |  |  |  |
| 1 | BR:Vic1:22, BR:Vic2:22, BR:Vic7:22, BR:Vic4.2:22 | 50 | 1401 | BR:Vic3:22 | Unknow | **R**GBMCST | 4.096 x 10^-05^ |  |
| 2 | BR:Coi153:14, BR:Vic13:10,  BR:Coi1:23, BR:Mur1:23, BR:Vic6:22, BR:Vic5:22 | 55 | 2026 | BR:Vic20:10 | Unknow | RG**B**MCST | 2.843 x 10^-03^ |  |
| 3 | BgV06A.1.C80 | 2650 | 1593 | BR:Coi157:14 | Unknow | R**G**BMCST | 4.263 x 10^-05^ |  |
| 4 | BR:Vic3:22, BR:Coi3:23, BR:Coi2:23, BR:Vic8:22,  BR:Vic7:22, BR:Vic2:22, BR:Vic4.1:22, BR:Vic4.2:22, BR:Vic1:22 | 1589 | 50 | BR:Coi157:14 | BR:Mac1:23 | **R**MCT | 3.815 x 10^-02^ |  |
| 5 | BR:Coi32.2:13, BR:Coi32.1:13 | 1841 | 2656 | BR:Coi152.1:14 | Unknow | MCS**T** | 1.457 x 10^-02^ |  |

^*^Numbering starts at the first nucleotide after the cleavage site at the origin of replication and increases clockwise
**^#^**R, Rdp; G, Geneconv; B, Boostcan; M, Maxichi; C, Chimaera; S, SisterScan; T, 3Seq
^&^The reported *p*-value is from the method in bold underlined, and is the lowest *p*-value calculated for the featured event

**Suppl. Table S6** Results of neutrality tests and mean ratios of non-synonymous to synonymous substitutions (dN/dS) for each gene of Blainvillea yellow spot virus (*Begomovirus blainvilleae*) isolates classified into five subpopulations^#^ by Discriminant Analysis of Principal Components (DAPC).

| **Subpopulation** | **Gene** | **Tajima's D^&^** | **Fu and Li's D*** | **Fu and Li's F*** | **dN/dS** |
| --- | --- | --- | --- | --- | --- |
| pop 1 | *Rep* | 0.37522 | 0.56715 | 0.56279 | 0.169 |
|  | *TrAP* | 1.18059 | 1.46717 | 1.48929 | - |
|  | *REn* | 0.33839 | 0.51052 | 0.49804 | 0.132 |
|  | *AC4* | -^$^ | - | - | - |
|  | *CP* | 1.13697 | 1.43971 | 1.49228 | 0.0847 |
| pop 2 | *Rep* | 0.5746 | 0.60436 | 0.65624 | 0.181 |
|  | *TrAP* | 0.32163 | 0.3419 | 0.36558 | 0.357 |
|  | *REn* | 0.01296 | 0.08022 | 0.07133 | 0.142 |
|  | *AC4* | -0.12471 | -0.1377 | -0.14477 | 0.575 |
|  | *CP* | 0.28268 | 0.54081 | 0.53158 | 0.00984 |
| pop 3 | *Rep* | -0.72617 | -0.02947 | -0.24858 | 0.153 |
|  | *TrAP* | -0.92501 | -0.4309 | -0.17465 | 0.626 |
|  | *REn* | -0.9475 | -0.59439 | -0.78858 | 0.195 |
|  | *AC4* | -0.76576 | -0.80311 | -0.90526 | 1.26 |
|  | *CP* | 0.20077 | 0.69196 | 0.64165 | 0.0277 |
| pop 4 | *Rep* | -1.01471 | -0.74684 | -0.91082 | 0.168 |
|  | *TrAP* | -0.28488 | 0.0081 | -0.07014 | 0.917 |
|  | *REn* | -0.66122 | -0.57771 | -0.67027 | 0.248 |
|  | *AC4* | -1.14944 | -1.03151 | -1.17694 | 1.24 |
|  | *CP* | -1.01516 | -0.98879 | -1.11705 | 0.0325 |
| pop 5 | *Rep* | -1.24264 | -1.6965 | -1.81821 | 0.162 |
|  | *TrAP* | -0.84373 | -0.76933 | -0.91901 | 0.394 |
|  | *REn* | -1.53543 | -2.16965 | -2.30463 | 0.320 |
|  | *AC4* | -1.43743 | -1.94567 | -2.08695 | 0.894 |
|  | *CP* | -0.90684 | -0.99903 | -1.13098 | 0.0318 |
| TOTAL | *Rep* | -0.5724 | -0.48826 | -0.62346 | 0.168 |
|  | *TrAP* | -0.5994 | 0.35722 | -0.00338 | 0.518 |
|  | *REn* | -0.98395 | -0.9895 | -1.18059 | 0.223 |
|  | *AC4* | -0.8372 | -1.1942 | -1.2682 | 0.874 |
|  | *CP* | -0.79404 | -0.02372 | -0.37902 | 0.0279 |

**^*^**pop1 corresponds to variant A, pop2 to variant D, pop4 to variant B, and pop5 to variant C. pop3 isolates were not classified into a variant

**^&^**No values of neutrality were significant to reject the null hypothesis of selective neutrality at *p* < 0.05
^$^Value cannot be calculated because the sequences are too similar

**Suppl. Table S7.** Number of amino acid sites under positive and negative selection* for each gene of Blainvillea yellow spot virus (*Begomovirus blainvilleae*) isolates classified into five subpopulations^#^ by Discriminant Anaçysis of Principal Components (DAPC).

|  |  | **SLAC** | | **FEL** | | **FUBAR** | |
| --- | --- | --- | --- | --- | --- | --- | --- |
| **Subpopulation** | **Gene** | **Positive  selection** | **Negative selection** | **Positive selection** | **Negative selection** | **Positive selection** | **Negative selection** |
| pop1 | *Rep* | 0 | 0 | 0 | 20 | 2 | 19 |
|  | *TrAP* | 0 | 2 | 0 | 3 | 1 | 4 |
|  | *REn* | 0 | 1 | 0 | 5 | 0 | 6 |
|  | *AC4* | 0 | 0 | 0 | 1 | 1 | 2 |
|  | *CP* | 0 | 0 | 0 | 23 | 0 | 32 |
| pop2 | *Rep* | 0 | **4** | 0 | 27 | 0 | 24 |
|  | *TrAP* | 0 | 2 | 0 | 8 | 2 | 6 |
|  | *REn* | 0 | 2 | 0 | 14 | 0 | 6 |
|  | *AC4* | 0 | 0 | 0 | 1 | 2 | 2 |
|  | *CP* | 0 | 4 | 0 | 19 | 0 | 22 |
| pop3 | *Rep* | 0 | 0 | 0 | 5 | 1 | 9 |
|  | *TrAP* | 0 | 0 | 0 | 0 | 1 | 0 |
|  | *REn* | 0 | 0 | 0 | 5 | 0 | 4 |
|  | *AC4* | 0 | 0 | 0 | 0 | 0 | 1 |
|  | *CP* | 0 | 1 | 0 | 23 | 0 | 20 |
| pop4 | *Rep* | 0 | 0 | 0 | 3 | 0 | 4 |
|  | *TrAP* | - | - | - | - | - | - |
|  | *REn* | 0 | 0 | 0 | 0 | 0 | 1 |
|  | *AC4* | - | - | - | - | - | - |
|  | *CP* | 0 | 0 | 0 | 4 | 0 | 9 |
| pop5 | *Rep* | 0 | 7 | 1 | 36 | 3 | 20 |
|  | *TrAP* | 0 | 2 | 0 | 6 | 2 | 5 |
|  | *REn* | 0 | 2 | 0 | 7 | 0 | 4 |
|  | *AC4* | 0 | 1 | 0 | 3 | 1 | 2 |
|  | *CP* | 0 | 9 | 0 | 23 | 0 | 21 |
| TOTAL | *Rep* | **1** | **45** | 2 | 89 | 2 | 75 |
|  | *TrAP* | 0 | 8 | 3 | 16 | 9 | 12 |
|  | *REn* | 0 | 11 | 0 | 24 | 1 | 18 |
|  | *AC4* | 0 | 1 | 1 | 4 | 5 | 2 |
|  | *CP* | 0 | 51 | 0 | 92 | 0 | 89 |

*Positive and negative selection at sites with a significance level of 0.1

^#^pop1 corresponds to variant A, pop2 to variant D, pop4 to variant B, and pop5 to variant C. pop3 isolates were not classified into a variant

**Suppl. Table S8.** Interspecific recombination analysis performed using RDP5 to detect recombination among Blainvillea yellow spot virus (*Begomovirus blainvilleae*, BlYSV) and nine other begomoviruses.

| **Dataset** | **BlYSV as recombinant** | **Recombinants** | **Method** | ***P-value*** |
| --- | --- | --- | --- | --- |
| All BlYSV isolates | No | - | - | - |
| Only Var. A | No | - | - | - |
| Only Var. B | No | - | - | - |
| Only Var. C | No | - | - | - |
| Only Var. D | No | - | - | - |
| Only unclassified isolates | Yes | BgV06A.1.C80, BgV06A.1.C81,  BR:Rla4:10, BR:Rla5:10,  BR:Rla6:09, BR:Rla1:23,  BR:Rla2:23, BR:Ral3:20, BR:Ral4:20, BR:Tou3:23, BR:Coi32.1:13, BR:Coi32.2:13, ALM13_2X (MacYSV*) , BR:Agf1:10 (MacYSV), BR:Crb1:11 (MacYSV) , BR:PE:CAU:22_2 (MacYSV) | RGBMCS3 | 6.82 x 10^-07^ |

* MacYSV indicates an isolate of Macroptilium yellow spot virus (*Begomovirus macroptilimaculae*).

Reliable breakpoint positions or parental lineages could not be confidently inferred.


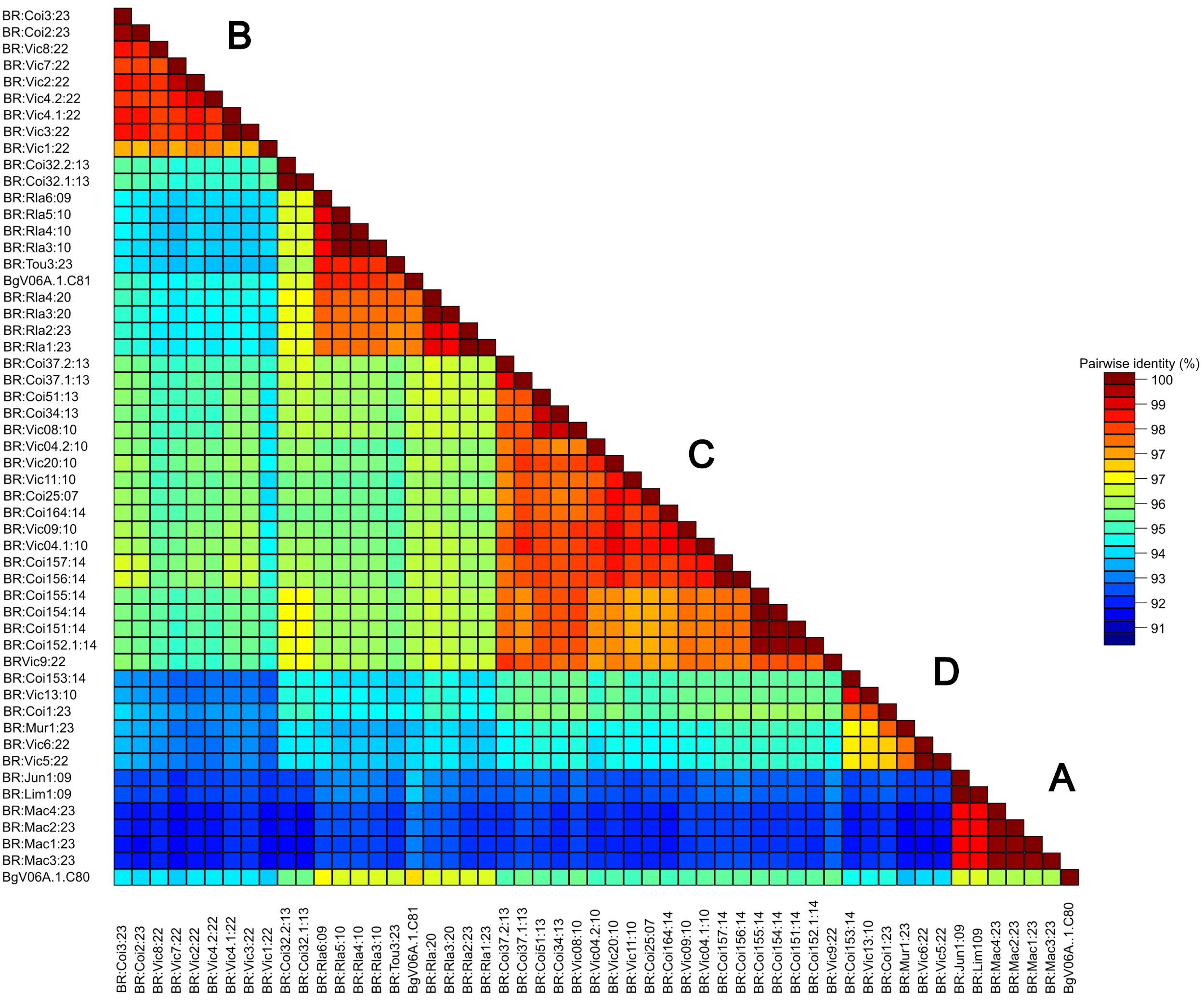


**Suppl. Figure S1.** Pairwise comparison matrix of Blainvillea yellow spot virus (*Begomovirus blainvilleae*) DNA-A sequences prepared with Sequence Demarcation Tool (SDT) v. 1.3. Isolates with nucleotide identity >96% were classified as belonging to the same variant (indicated by the uppercase letter).


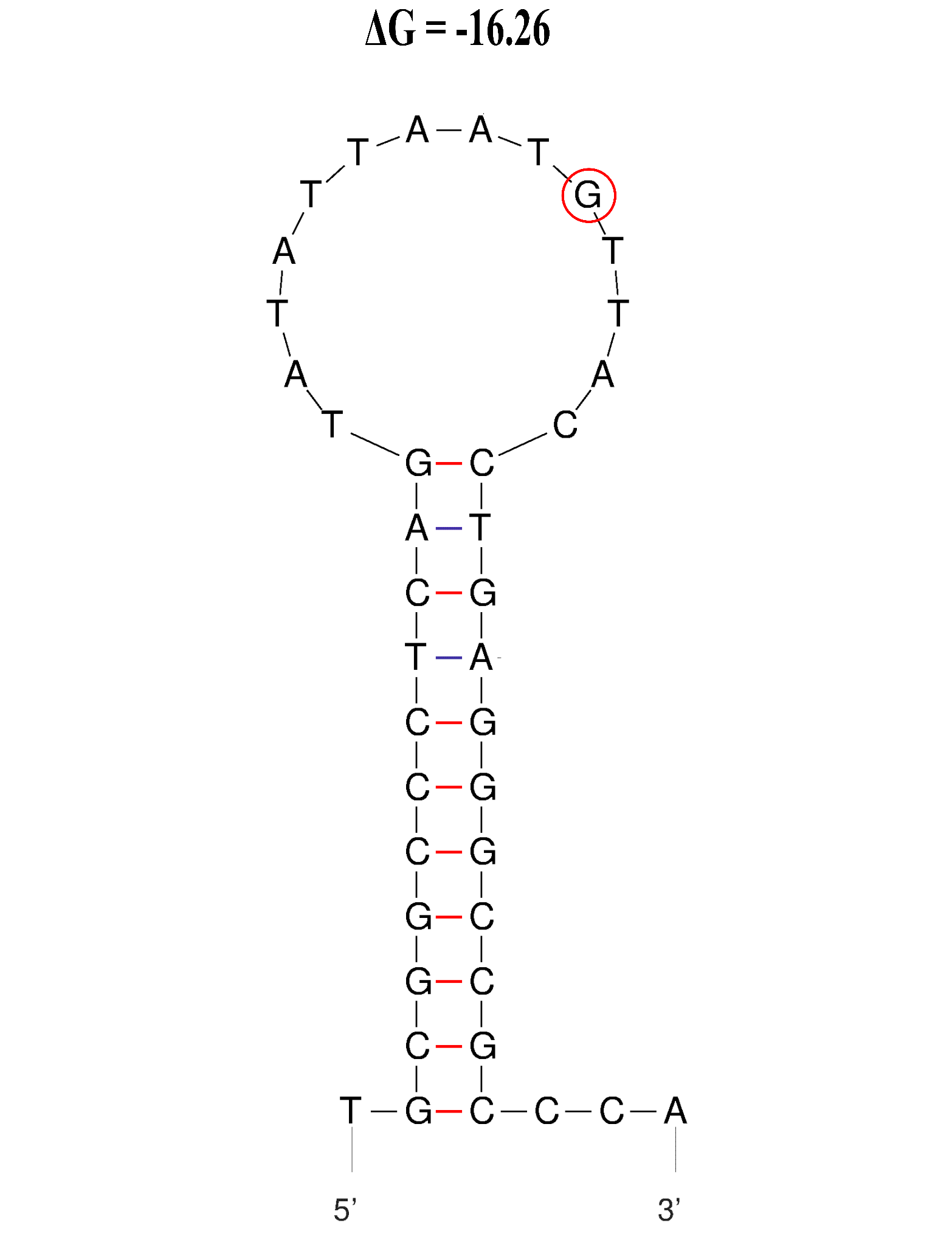


**Suppl. Figure S2.** Secondary structure of the origin of replication of the Blainvillea yellow spot virus (*Begomovirus blainvilleae*) isolate BR:Mac1:23, with the alternative nonanucleotide motif TAATGTTAC. The structural analysis was performed using the Mfold web server. The red circle highlights the A2667G substitution.


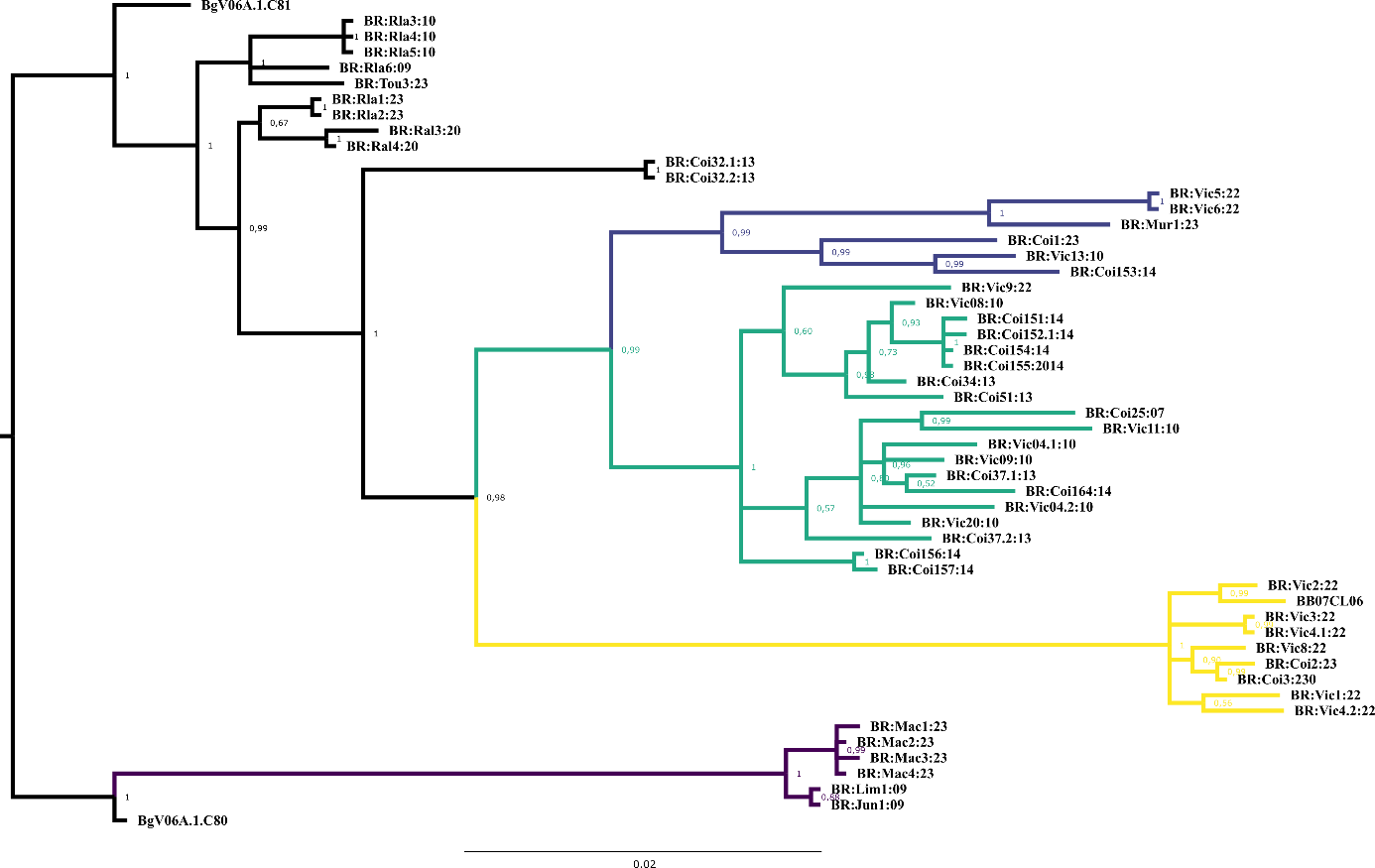


**Suppl. Figure S3.** Phylogenetic tree based on the *Rep* gene nucleotide sequence of Blainvillea yellow spot virus (*Begomovirus blainvilleae*) isolates. The tree was constructed using Bayesian inference, with posterior probability values indicated at each branch. Branches are color-coded according to the variants in which the isolates are classified: purple, variant A; yellow, variant B; green, variant C; blue, variant D; black, isolates that were not classified into variants.


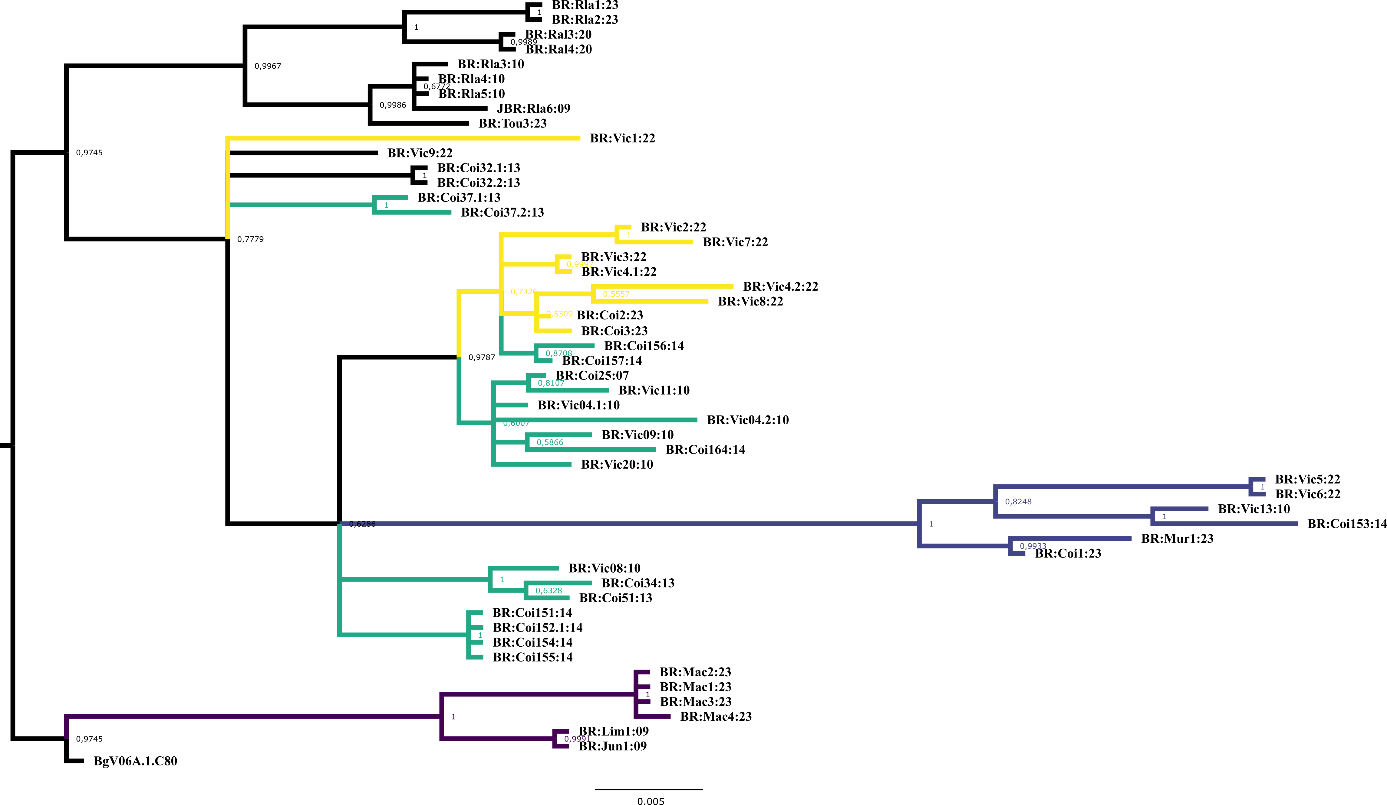


**Suppl. Figure S4.** Phylogenetic tree based on the *CP* gene nucleotide sequence of Blainvillea yellow spot virus (*Begomovirus blainvilleae*) isolates. The tree was constructed using Bayesian inference, with posterior probability values indicated at each branch. Branches are color-coded according to the variants in which the isolates are classified: purple, variant A; yellow, variant B; green, variant C; blue, variant D; black, isolates that were not classified into variants.


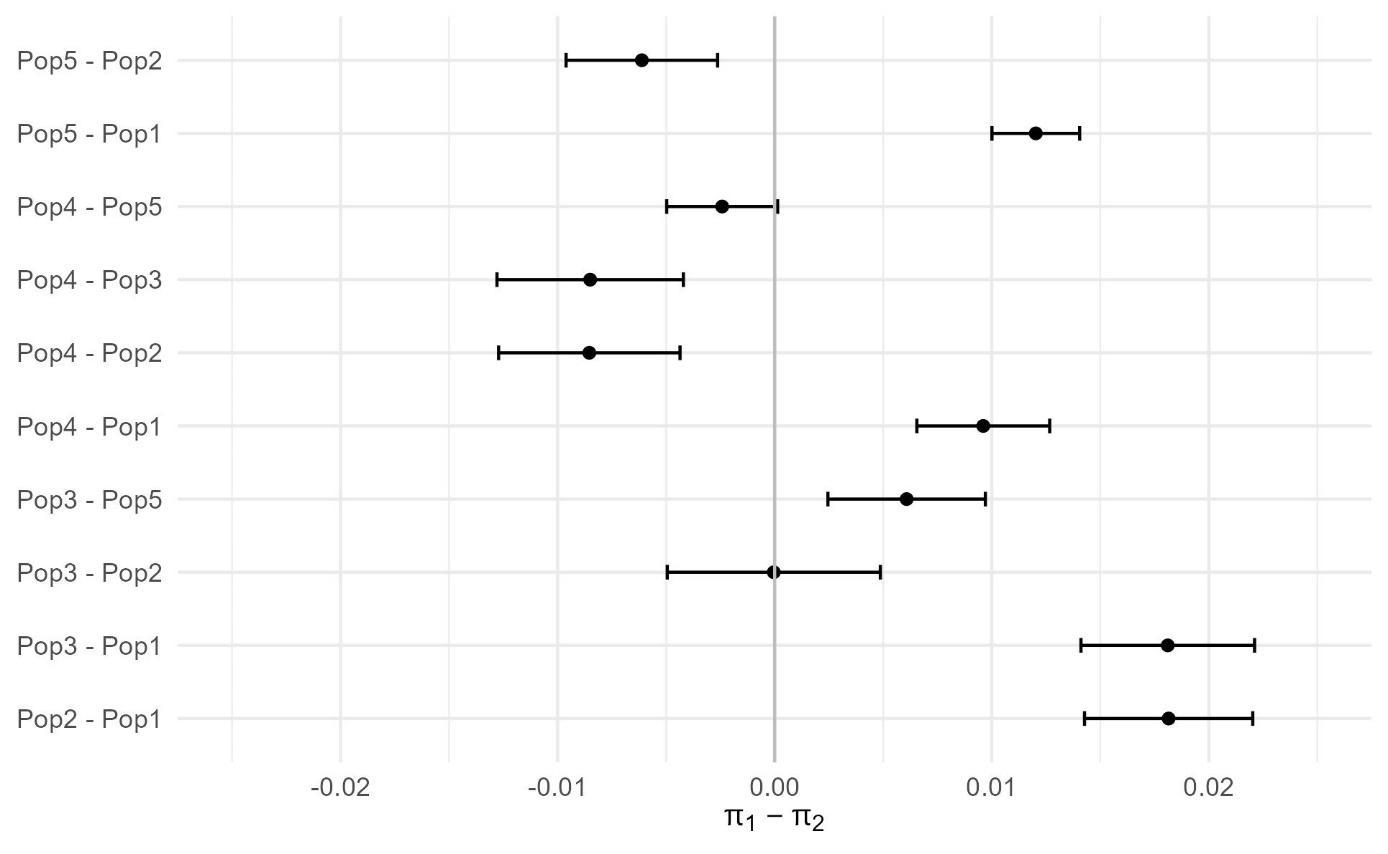


**Suppl. Figure S5.** Assessment of the statistical significance in the differences of nucleotide diversity (π) values among the five subpopulations of Blainvillea yellow spot virus (*Begomovirus blainvilleae*) as determined by Discriminant Analysis of Principal Components (DAPC). pop1 corresponds to variant A, pop2 to variant D, pop4 to variant B, and pop5 to variant C. pop3 isolates were not classified into a variant. The differences in π where the confidence interval (bars) overlaps the value 0 are not statistically significant.


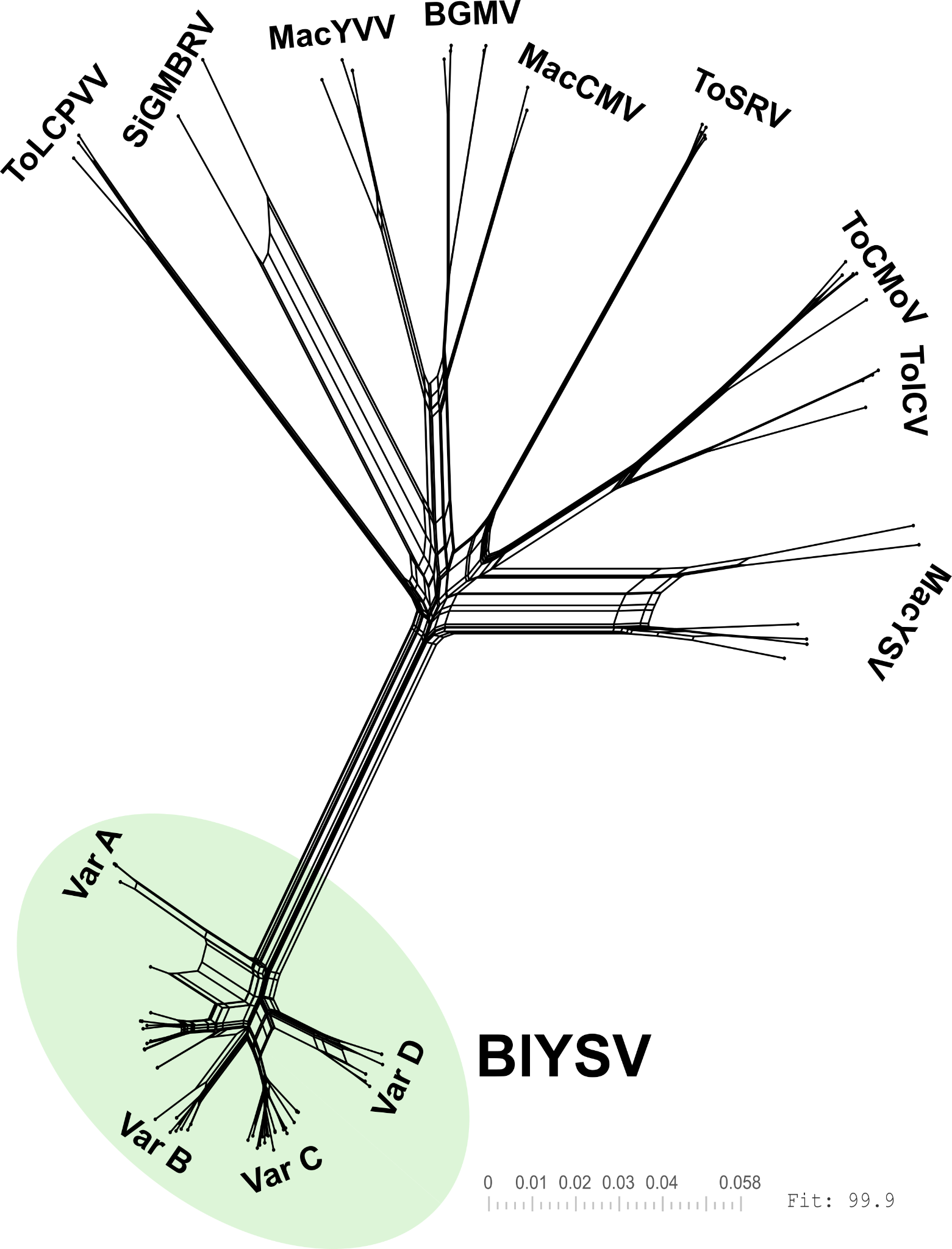


**Suppl. Figure S6.** NeighborNet network constructed in SplitsTree based on complete DNA-A sequences of Blainvillea yellow spot virus (*Begomovirus blainvilleae*, BlYSV) and nine other begomoviruses. The analysis includes Sida golden mosaic Brazil virus (*B. sidaureibrazilense,* SiGMBRV), tomato leaf curl purple vein virus (*B. solanumviolavenae*, ToLCPVV), bean golden mosaic virus (*B. costai,* BGMV), Macroptilium yellow spot virus (*B. macroptilimaculae*, MacYSV), tomato interveinal chlorosis virus (*B. solanumintervenae*, ToICV), Macroptilium common mosaic virus (*B. macroptilicommunis*, MacCMV), Macroptilium yellow vein virus (*B. macroptilivenae*, MacYVV), tomato severe rugose virus (*B. solanumseverugosi,* ToSRV), and tomato chlorotic mottle virus (*B. solanumpallidivariati*, ToCMoV).
